# Reducing ventriculoperitoneal shunt infection with intraoperative glove removal

**DOI:** 10.1017/ice.2022.70

**Published:** 2023-02

**Authors:** Konrad W. Walek, Michal Rajski, Rahul A. Sastry, Leonard A. Mermel

**Affiliations:** 1 Department of Neurosurgery, Warren Alpert Medical School of Brown University, Providence Rhode Island; 2 Department of Medicine, Warren Alpert Medical School of Brown University, Providence Rhode Island; 3 Department of Epidemiology and Infection Control, Rhode Island Hospital, Providence, Rhode Island; 4 Division of Infectious Diseases, Rhode Island Hospital, Providence, Rhode Island

## Abstract

**Background:**

Contamination of ventriculoperitoneal shunts (VPS) by cutaneous flora, particularly coagulase-negative staphylococci, is a common cause of shunt infection and failure, leading to prolonged hospital stay, higher costs of care, and poor outcomes. Glove contamination may occur during VPS insertion, increasing risk of such infections.

**Methods:**

We performed a systematic search of the PubMed database for studies published January 1, 1970, through August 31, 2021 that documented VPS infection rates before and after implementing a practice of double gloving with change or removal of the outer glove immediately prior to shunt insertion.

**Results:**

Among 272 reports screened, 4 were eligible for review based on our inclusion criteria. The incidence of VPS infection was reduced in all 4 quasi-experimental studies with an aggregate incidence of VPS infection of 11.8% before the change in intraoperative protocol and 4.9% after protocol change. One study documented reduced hospital stay with this change in protocol.

**Conclusion:**

The risk of VPS infection is reduced by removal or replacement of the outer surgical gloves immediately prior to intraoperative insertion of a VPS as part of an infection control bundle.

Ventriculoperitoneal shunt (VPS) insertion is a common neurosurgical procedure and firstline treatment for chronic hydrocephalus. Incidence of VPS infections varies from <1% to 20%,^1–19^ with reported mortality rates of 1%–22%.^8,19–21^ Many VPS infections are due to skin flora, particularly *Staphylococcus epidermidis* and *S. aureus*.^1,2,4,5,7,10–15,17,18^ VPS infection risk has been associated with male sex, low socioeconomic status, young age (≤18 years old), diabetes, previous VPS revision or infection, type of hydrocephalus, myelomeningocele, use of intraoperative single-glove technique, intraoperative duration of VPS insertion, number of surgeons and experience of surgeons performing VPS insertion, and postoperative CSF leak.^1–3,7–14,18,19^

Management of VPS infection entails shunt removal and antibiotic treatment, and such infections may lead to shunt failure, prolonged hospitalization, neurological disability, increased hospital cost, and higher mortality.^1,4–6,8,11,12,15,18–22^ Strategies used to prevent VPS infection include preoperative use of chlorhexidine shampoo, cutaneous antisepsis (eg, use of chlorhexidine or povidone-iodine), rigorous aseptic technique, limiting shunt contact with the patient’s skin during insertion, using instruments to handle the shunt intraoperatively, a no-shave policy, hematoma prevention, and laparoscopic VPS placement.^3–8,10,13,16,23–25^

VPS infection risk can be significantly reduced by double gloving for shunt insertion and manipulation.^1,2,6,8,23,24^ Moreover, removing or changing the outer glove immediately prior to handling the VPS may further reduce infection risk, but the significance and magnitude of this effect has thus far been variable.^3,5,9,13^ In this literature review, we have summarized available evidence for surgical personnel to remove or change their outer gloves prior to handling shunt instrumentation.

## Methods

**Fig. 1:**
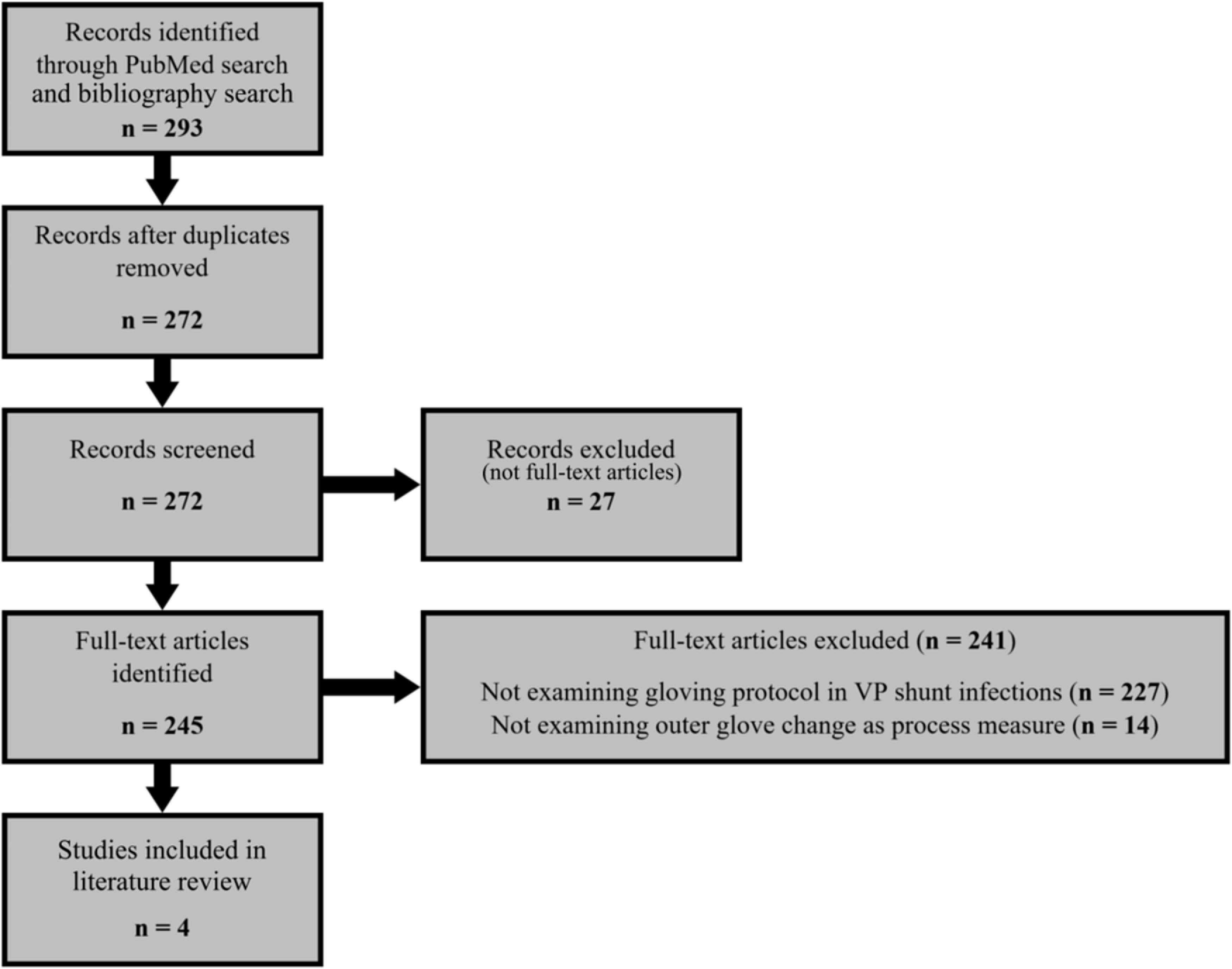
PRISMA flow diagram. VP, ventriculoperitoneal.

We performed a systematic search of the PubMed database for studies of institutions documenting VPS infection rates before and after standardizing double gloving with removal or change of the outer glove immediately before shunt insertion using the Preferred Reporting Items for Systematic Reviews and Meta-Analysis (PRISMA) guidelines.^26^ PubMed was used to search for articles in any language published January 1, 1970, through August 31, 2021 using the following search terms: surgical infection glove change (261 articles), shunt glove change (9 articles), and shunt infection glove (17 articles). Bibliographies of articles investigating outer-glove removal or change during CSF shunt placement were also searched, producing an additional 6 articles not previously identified, leading to a total of 272 identified articles after 21 duplicates were removed. The methods sections of all full-text articles were assessed for eligibility. Studies were included if a control group was used to compare the CSF shunt infection rates following placement with or without intraoperative removal or change of the outer pair of double gloves immediately prior to handling the shunt before insertion. Studies were excluded if there was no comparison of outer-glove removal or change to no glove removal or change, or if only qualitative comparative results were provided. The primary outcome measure for all included articles was a VPS infection. Of the 245 full-text articles identified among the 272 identified records, 18 addressed glove protocol in VPS placement. Of these 18 records, 14 were ineligible for not specifically documenting intraoperative outer glove removal or change immediately before VPS manipulation as a process measure. Ultimately, 4 studies met the inclusion criteria^3,5,9,13^ (Fig. 1). Meta-analysis was not performed given the low number of studies and heterogeneity between the studies.

## Results

We identified 4 quasi-experimental, single-center studies involving 934 patients of all ages.^3,5,9,13^

One study prospectively assessed VPS infection rates in a pediatric population (aged <18 years) undergoing shunt insertion surgery after implementation of an intraoperative infection control protocol that included removal of the outer gloves prior to VPS insertion, completing the procedure with a single glove layer.^3^ The second study retrospectively assessed VPS infection rates in 2 consecutive cohorts of a neonatal population (aged <1 month) undergoing shunt insertion surgery.^5^ The authors used an intraoperative infection prevention bundle in for the second cohort with the addition of an adhesive sheet to the skin after draping and double gloving with the removal of the outer-glove layer before handling the shunt. The procedure was completed with a single glove layer. The third study retrospectively assessed VPS infection rates in 2 consecutive cohorts of an adult population (aged >18 years) undergoing shunt insertion surgery before and after implementation of an outer-glove change protocol.^9^ These investigators used an intraoperative infection prevention bundle for both cohorts; however, in the second cohort, the investigators added a change of the second glove layer before handling the shunt materials. The final study retrospectively assessed VPS infection rates in 2 consecutive cohorts adult patients undergoing shunt insertion surgery before and after implementation of an intraoperative infection prevention bundle that included replacement of the outer glove layer prior to handling shunt materials.^13^

Three studies included outer-glove change or removal as part of an infection control bundle; 1 study investigated outer-glove change without other concomitant infection control interventions. Across the 4 studies, the aggregate incidence of VPS infection was 11.8% before the change in intraoperative protocol (55 infections among 465 patients) and 4.9% after the protocol change (23 infections among 469 patients). Of the 4 studies, 2 reported a significant difference in the incidence of VPS infections with and without intraoperative outer-glove removal prior to VPS insertion (Table 1), regardless of duration of surgery, combined surgery (eg, with cranioplasty), or immunosuppressed status (Table 1).^5,9,13^ Outer-glove change was also associated with shorter hospital length of stay.^5^ A significant decrease in the likelihood of VPS infection was also observed with outer-glove change at 6-month follow-up in a subgroup of patients who underwent first-time VPS insertion after adjusting for the cause of hydrocephalus^9^ (Table 1).

**Table 1.** Incidence of Ventriculoperitoneal Shunt (VPS) Infections Before and After Standardized Intraoperative Outer-Glove Change or Removal

Reference	Study Design	Patient Age	Implementation	Outcome Measures and Shunt Infection Rates		
				Incidence of VPS Infections With Double Gloves, Without Outer-Glove Removal or Change	Incidence of VPS Infections With Double Gloves, With Outer-Glove Removal or Change	Follow-Up (Months)
Prusseit et al^3^	Prospective, quasi-experimental, single center	<18 y	Removing outer gloves prior to VPS insertion^a^	17% (17/100)	1% (1/115)^b^	4–70
Kestle et al^5^	Retrospective, quasi-experimental, single center	<1 mo	Removing outer gloves prior to VPS insertion^a^	15% (8/54)	4% (2/57)^c^	6
Omrani et al^9^	Retrospective, quasi-experimental, single center	≥18 y	Replacing outer gloves prior to VPS insertion	11% (21/187)	8% (20/245)^d^	6–12
Mallucci et al^13^	Retrospective, quasi-experimental, single center	≥18 y	Replacing outer gloves prior to implant insertion^a^	7% (9/124)	0% (0/52)^e^	36

a Glove change/removal was part of an infection control bundle.

b OR, 0.04; 95% CI, 0.01–0.33; *P* = .002.

c OR, 0.24; 95% CI, 0.06–0.94; *P* = 0.046.

d OR, 0.90; 95% CI, 0.4.0–2.06; *P* = 0.807; at 6 month follow-up OR 0.10: 95% CI, 0.01–1.01; *P* = .050.

e OR, 0.12; 95% CI, 0.01–2.03; *P* = 0.140.

## Discussion

A significant reduction in risk of VPS infection was achieved with a change from single gloves to double gloves during VPS insertion.^1,2,24^ In our review of the literature, intraoperative outer-glove change or removal prior VPS insertion, particularly as part of an infection control bundle, appears to further reduce risk of VPS infections. Other strategies for preventing VPS infection have been proposed,^2–7,9,10,13–15,17,18^ including use of intraventricular and topical vancomycin,^17^ antibiotic-containing sutures,^8,23^ and antimicrobial-impregnated shunts.^7,14^ Some of these strategies have demonstrated feasibility of systematic implementation with consistent and significant reductions in VPS infections.^8,16,27^ However, given the varying quality of evidence underlying these strategies, as well as provider preference, implementation is not standardized across US hospitals.^23,24,28^ Use of antimicrobial-impregnated catheters has been shown to reduce VPS infections,^4,7,8,11,14,16^ but cost of implementation has been cited as a barrier to implementation, particularly in resource-limited environments.^11,16,28^

Glove change before handling vascular grafts and cardiac implantable electronic devices has been demonstrated to reduce glove contamination.^29,30^ Systematic intraoperative glove changes during orthopedic surgery reduces the frequency of occult perforations and bacterial loading of glove surfaces.^31–34^ Substantial glove contamination has also been demonstrated intraoperatively before handling a VPS for insertion.^1,35,36^ Some authors have suggested frequent glove changes throughout surgical procedures, at least every 20–90 minutes and at critical points (eg, after draping, before handling of instrumentation, and prior to wound closure).^37,38^ In 1 study, standardizing glove change 1 hour after initiating surgery resulted in a 10% absolute reduction in glove contamination.^35^ During VPS insertion, one group of investigators found that contamination of sterile gloves occurred within 15 minutes of donning and recommended that ‘a simple measure would be to change the outer pairs of gloves before handling of the shunt material during surgery.’^36^ Unsurprisingly, outer-glove change prior to handling instrumentation during lumbar fusion led to an 86% reduction in postoperative infections.^39^ Nevertheless, more data are needed with glove change alone to prove its utility as a standard of care for all procedures involving implantation of permanent devices.

Our review has a number of limitations. Publication bias is possible due to the small number of studies that met our inclusion criteria. Heterogeneity of the studies was high, and the studies were underpowered. One study assessed only intraoperative glove removal or change,^9^ whereas the 3 others assessed intraoperative glove change as part of an infection prevention bundle.^3,5,13^ In the lone study that assessed glove change alone,^9^ the reduction in VPS infection rate only reached statistical significance at a 6-month follow-up. As such, the attributable effect of outer-glove removal alone is likely contributory to lower infection rates, but further research is needed to unequivocally confirm the impact of this preventative strategy.

In conclusion, reducing risk of a VPS infection may be achieved through a low-cost protocol of standardizing outer-glove removal or change as part of an infection control bundle prior to handling of the shunt during intraoperative insertion. Replacing the outer glove may be preferable in the event that the inner glove was contaminated due to unsuspected outer-glove perforation during a surgical procedure or during outer-glove removal. This intervention does not require purchase of new equipment, is easy to implement, and can be utilized in resource limited and well-resourced settings.
